# Transrectal high‐intensity focused ultrasound (HIFU) for management of rectosigmoid deep infiltrating endometriosis: results of Phase‐I clinical trial

**DOI:** 10.1002/uog.21937

**Published:** 2020-09-01

**Authors:** C.‐A. Philip, S. Warembourg, M. Dairien, C. Lefevre, A. Gelet, F. Chavrier, N. Guillen, H. Tonoli, E. Maissiat, C. Lafon, G. Dubernard

**Affiliations:** ^1^ Gynecology Department Croix‐Rousse University Hospital Hospices Civils de Lyon, Lyon France; ^2^ Claude Bernard Lyon 1 University, University of Lyon Lyon France; ^3^ LabTAU, INSERM (Unit 1032), Centre Léon Bérard, Lyon 1 University F‐69003 Lyon France; ^4^ EDAP‐TMS Company Vaulx‐en‐Velin France; ^5^ Radiology Department Croix‐Rousse University Hospital Hospices Civils de Lyon, Lyon France

**Keywords:** DIE, digestive endometriosis, HIFU treatment, minimally invasive surgery, rectosigmoid endometriosis, sonography

## Abstract

**Objectives:**

Deep infiltrating endometriosis (DIE) of the rectosigmoid is associated with painful symptoms. When medical treatment is ineffective, surgical resection remains the standard treatment, despite significant risk of adverse events. High‐intensity focused ultrasound (HIFU) is a minimally invasive ablative procedure. Focal One® is a transrectal HIFU (TR‐HIFU) device used in prostate cancer treatment. The primary objective of this study was to confirm the feasibility of treatment with TR‐HIFU in patients presenting with posterior DIE with rectosigmoid involvement. We also assessed its safety and clinical efficacy in this context.

**Methods:**

This was a non‐controlled, prospective, Phase‐I clinical trial in a French University Hospital which is a multidisciplinary center for management of endometriosis. Included were patients older than 25 years, without plans to conceive within 6 months, who presented with a single lesion of posterior DIE, with rectosigmoid invasion, after failure of hormonal therapy. All lesions were assessed preoperatively using transvaginal sonography and magnetic resonance imaging. Patients completed questionnaires on gynecological and intestinal symptoms (similar to a visual analog scale (VAS)), and on quality of life (Medical Outcomes Study 36‐item short‐form survey (SF‐36) and, for the second half of patients recruited, symptom scoring system for constipation (KESS), female sexual function index (FSFI) and endometriosis health profile short‐version score (EHP‐5)), before, and at 1, 3 and 6 months after, TR‐HIFU treatment with a Focal One real‐time ultrasound‐guided HIFU device.

**Results:**

Twenty‐three consecutive patients were included in the study between September 2015 and October 2019. All 23 lesions were visualized, giving a detection rate of 100%. Twenty lesions were treated (‘feasibility rate’, 87.0%): in 13 the whole lesion was treated and in seven the lesion was treated partially. The mean duration of the TR‐HIFU procedure was 55.6 min. We observed a significant improvement in VAS score at 6 months, with differences relative to preoperative scores as follows, for: dysmenorrhea (–3.6, *P* = 0.004), dyspareunia (–2.4, *P* = 0.006), diarrhea (–3.0, *P* = 0.006), constipation (–3.0, *P* = 0.002), dyschezia (–3.2, *P* = 0.003), false urge to defecate (–3.3, *P* = 0.007), posterior pelvic pain (–3.8, *P* = 0.002) and asthenia (–3.8, *P* = 0.002). There was also a significant improvement in the SF‐36 score, with an increase at 6 months relative to the preoperative score in both the physical component summary (+ 9.3%, *P* = 0.002) and mental component summary (+ 10.9%, *P* = 0.017). No major complications occurred during or after any procedure.

**Conclusions:**

TR‐HIFU therapy for posterior DIE is feasible. If its efficacy and safety are confirmed, it could be a minimally invasive alternative to surgery for the treatment of rectosigmoid endometriosis. © 2019 Authors. *Ultrasound in Obstetrics & Gynecology* published by John Wiley & Sons Ltd on behalf of International Society of Ultrasound in Obstetrics and Gynecology.

**CONTRIBUTION**

*What does this work add to what is already known?*

We report for the first time that transrectal high‐intensity focused ultrasound (HIFU) for therapy for rectosigmoid endometriosis is feasible. No major complication was observed after 20 procedures. We report a significant effect on gynecological and digestive symptoms. Morphological effects remain to be demonstrated.

*What are the clinical implications of this work?*

Transrectal HIFU could be a minimally invasive alternative to surgery in rectal endometriosis. It could decrease the incidence of short‐ and long‐term complications of this pathology. Further studies are required to demonstrate its clinical efficiency and safety.

## INTRODUCTION

Endometriosis affects 10% of women of childbearing age and between 2.2% and 81.7% of women with chronic pelvic pain^1^. Deep infiltrating endometriosis (DIE) is a form of endometriosis that infiltrates the muscularis of the abdominopelvic organs^2^. Invasion of the digestive tract accounts for between 3.8% and 37% of patients with DIE^3^. The most common intestinal location is the rectosigmoid, and this is responsible for specific painful symptoms^4^.

According to Roman^5^, surgical treatment for rectosigmoid DIE involves colorectal resection in 47% and conservative surgery in 53% of cases. Short‐term complications of surgery are dominated by rectovaginal fistulae and anastomotic leakage, which occur in 4.3% and 3.7% of cases, respectively^6^. To prevent these complications, an ileostomy is performed in 19.1% of cases^5^. Long‐term complications have been assessed less thoroughly in the literature but are just as debilitating and likely to persist over time^6^, ^7^, ^8^.

High‐intensity focused ultrasound (HIFU) was first described in 1942 by Lynn *et al*.^9^. This treatment releases a controlled burst of energy by focusing the ultrasound beam at a particular point to create a lesion in the tissue^10^. Juxtaposition in space of several lesions created by the HIFU beam leads to necrosis of the target volume. Treatment by HIFU is commonly used in the management of localized prostate cancers^11^, ^12^. Focal One®, a minimally invasive thermodestructive medical device designed for a transrectal approach, is the third generation of transrectal HIFU (TR‐HIFU) devices (EDAP‐TMS Company, Vaulx‐en‐Velin, France). It is a real‐time ultrasound‐guided HIFU device, and is also equipped with ultrasound–magnetic resonance imaging (MRI)‐fusion software. Several studies have reported the efficacy of HIFU for treating gynecological disorders such as uterine leiomyomas, adenomyosis, endometriosis parietal nodules and ectopic pregnancies on Cesarean scars^13^, ^14^, ^15^, ^16^, ^17^.

Our primary objective in this Phase‐I clinical trial was to evaluate the ability of the Focal One HIFU device to detect and target posterior DIE lesions with intestinal involvement. Our secondary objectives were to apply HIFU thermal ablation, evaluate morphological modifications of the endometriotic lesion in post‐therapeutic imaging scans, assess evolution of gynecological symptoms, intestinal symptoms and the patients' quality of life after treatment by HIFU, and to record potential postoperative complications.

## SUBJECTS AND METHODS

### Population

This was a prospective, non‐controlled, Phase‐I clinical trial, carried out at a French University Hospital which is a multidisciplinary center for management of endometriosis. Patients were recruited during a gynecological consultation by a physician expert in endometriosis.

Inclusion criteria were: presence of a single rectosigmoid DIE lesion, absence of any other endometriotic nodule (intestinal, urinary or ovarian) on imaging, persistence of symptoms despite hormonal treatment and therefore consideration for surgical management. Extension of the nodule into the retrocervical space and/or to the uterosacral ligaments was considered acceptable.

Initial exclusion criteria were: age below 35 years, inability to rule out plans to conceive, treatment with luteinizing‐hormone‐releasing‐hormone agonists in the preceding 3 months and presence of abnormal anorectal anatomy due to surgical history or congenital anomaly. After the first five cases, in which no major complications were experienced, l'Agence Nationale de Securité du Medicament (ANSM, the French National Drug Safety Agency) allowed us to expand the inclusion criteria to include age over 25 years and no plans to conceive in the 6‐month period following treatment (i.e. until after postoperative MRI).

The clinical trial was approved by the regional ethics committee (CPP SUD EST II, reference number 2014‐045‐2) under EudraCT 2014‐A01728‐39 and by ANSM. All patients provided written informed consent to participate.

### Preoperative assessment

**Figure 1:**
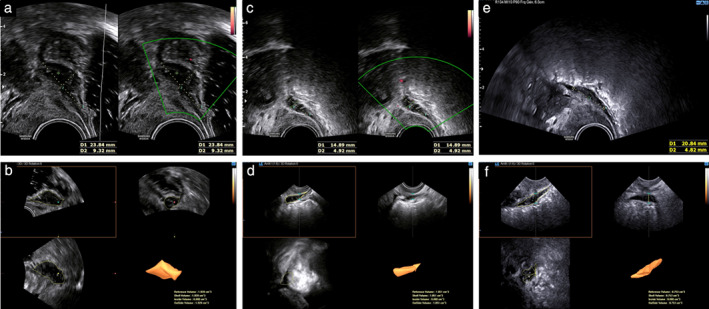
Transvaginal rectosonography, showing endometriotic rectal lesion in two‐dimensional (a,c,e) and three‐dimensional multiplanar (b,d,f) modes before (a,b) and 1 month (c,d) and 3 months (e,f) after transrectal high‐intensity focused ultrasound treatment. Intestinal endometriotic nodule appears as hypoechogenic lesion protruding into intestinal lumen. Volume is calculated using Virtual Organ Computer‐aided AnaLysis (VOCAL) mode (b,d,f). Initial diameters and volume (24 × 9 mm and 1.9 cm^3^) decreased over time (15 × 5 mm and 1.1 cm^3^ at 1 month and 20 × 5 mm and 0.75 cm^3^ at 3 months).

Preoperative assessment consisted of clinical and imaging examinations combining three‐dimensional rectosonography (3D‐RSG), i.e. transvaginal sonography with intrarectal contrast and 3D volume acquisition (Figure 1), and a pelvic MRI scan (without and then with gadolinium injection). Several major gynecologic societies recommend using a combination of transvaginal sonography and MRI as first‐line procedures for the diagnosis of DIE^18^, ^19^, ^20^. On 3D‐RSG, bowel involvement was suspected when a solid hypoechoic nodule was seen adhered to the serosal layer and infiltrating at least the outer intestinal muscularis^21^. On MRI, intestinal endometriotic lesions were seen as hypointense nodules, often poorly delimited, attached to the intestinal wall. T2 sequences enabled the precise extent and location of the lesions to be determined^22^. This assessment was used to locate the rectosigmoid lesion, to measure its dimensions and volume, to assess the degree of infiltration of the intestinal wall and to rule out the presence of other endometriotic lesions. The precise location of the lesion was identified on 3D‐RSG and then on MRI according to the anatomic classification of SNFGE, the French National Society of Gastroenterology^23^.

At inclusion, each patient completed a questionnaire, similar to a visual analog scale (VAS), which evaluated their gynecological and intestinal symptoms and has been validated for this indication^24^. They were also asked to fill out the Medical Outcomes Study 36‐item short‐form survey (SF‐36) to evaluate their quality of life^25^, ^26^, ^27^. SF‐36 provides an eight‐dimension profile which evaluates physical activity (includes: physical functioning, role limitation related to physical problems, physical (bodily) pain, overall general health, energy/fatigue (vitality), social functioning, role limitation related to emotional problems and emotional wellbeing (mental health)). A global physical score (physical component summary (PCS)) and a global mental score (mental component summary (MCS)) were also calculated from these different components.

The second half of the patients included were part of a second series in this Phase‐I clinical trial with renewed ethical and legal authorizations, as ANSM allowed us to treat only 10 patients in the first series. Patients of this second series completed additional questionnaires: the endometriosis health profile short‐version score (EHP‐5), symptom scoring system for constipation (KESS) and female sexual function index (FSFI)^28^, ^29^, ^30^, ^31^.

### Focal One

**Figure 2:**
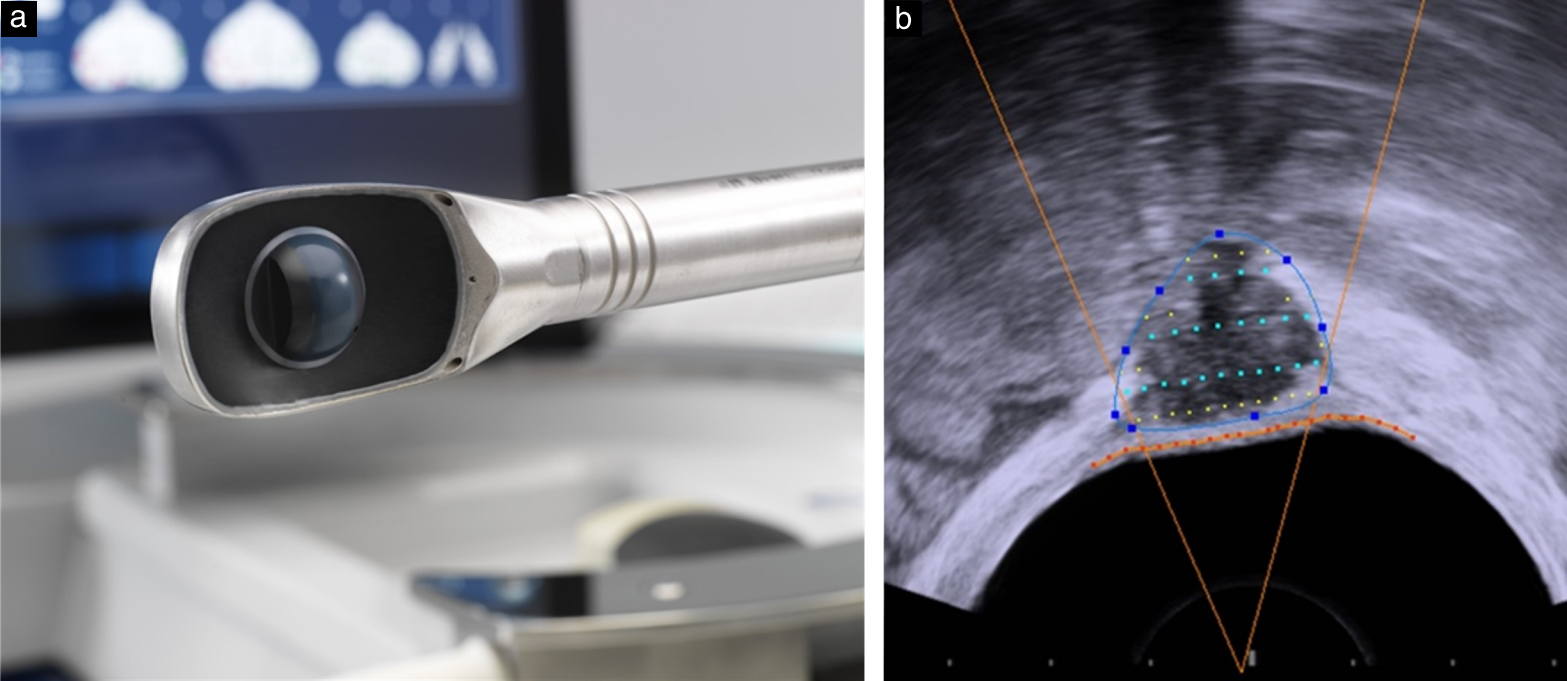
Endorectal probe (a) and mid‐procedure transrectal ultrasound image (b) from Focal One® device. (a) Endorectal dynamic focusing probe includes ultrasound therapy transducer (smooth black part surrounding ball) to deliver therapeutic ultrasound, and imaging transducer (ball, in center) to visualize and locate areas to be treated. (b) Endometriotic nodule is delimited manually (blue points). Green points are therapeutic ultrasound target points. Rectal wall and its safety margin are delimited by orange line; no HIFU shot was allowed within 3 mm of orange line.

Focal One (EDAP‐TMS, Vaulx‐en‐Velin, France) is a robotic computer‐controlled medical device used for TR‐HIFU ablation of prostate cancer. Available in Europe since June 2013 and in the USA since June 2018, it is a real‐time ultrasound‐guided HIFU device, equipped with ultrasound–MRI‐fusion software. The endorectal probe includes a HIFU transducer that delivers the therapeutic ultrasound and an ultrasound imaging transducer to visualize and locate areas to be treated (Figure 2a). The HIFU transducer is an annular array composed of 16 concentric rings. It is coupled to a multichannel amplifier, which, by shifting the phase of exciting electrical signals, moves the focal point of the ultrasonic beam electronically, without mechanical movement. This technology, designated ‘dynamic focusing’, allows the focal spot to be located between 32 and 67 mm from the center of the probe. The physical characteristics of the Focal One transducer are summarized in Table S1.

### Simulation and dose calculation

Numerical simulations were conducted in an INSERM laboratory specializing in HIFU, to determine which exposure conditions should be delivered by Focal One in order to ablate thermally a theoretical nodule between 1 and 11 cm^3^ in volume. A treatment sequence that allowed the best compromise between complete ablation and rectal preservation was established, by making certain assumptions regarding biological parameters of treated and surrounding tissues^32^. The precise nature of the acoustic characteristics of endometriotic nodules is uncertain due to the lack of published data and the likely variation of these characteristics according to the point in the menstrual cycle. The data were therefore extrapolated in part, based on the acoustic characteristics used in the treatment for fibroids^33^. Thus, taking into account these uncertainties, a value ranging between 2 and 60 Np.m^–1^.MHz^–1^ was used to simulate endometriotic nodule attenuation. We concluded that the HIFU nominal acoustic powers corresponding to 50% of that used in prostate cancer treatment was safe under all conditions (data not shown).

### Endpoints

The primary endpoint of this study was the feasibility of using this technique in the treatment of patients with rectosigmoid DIE, defined both by the ability to detect the endometriotic lesion and the ability to achieve all the conditions necessary to enable TR‐HIFU treatment (probe–nodule distance > 32 mm, no interposition of normal bowel and a safety margin of 3 mm between the target tissue and the intestinal mucosa). Regarding secondary endpoint, safety was assessed by systematic recording of all adverse events observed during follow‐up, classified according to the Clavien–Dindo classification^34^. Clinical efficacy was assessed using the imaging procedures and questionnaires described above.

### TR‐HIFU treatment protocol

Patients were hospitalized the day before the procedure in order to undergo bowel preparation. HIFU treatment was performed in an operating room with the patient in a right lateral decubitus position and under spinal anesthesia. The endorectal probe was covered with a balloon filled with an acoustic coupling cooling liquid (Ablasonic®, EDAP‐TMS) to facilitate transmission of the ultrasound beam and to maintain close contact with the rectal wall.

Two steps were used to assess the feasibility of detection. The first comprised locating and assessing the volume of the endometriotic lesion with the diagnostic transducer. Second, application of the HIFU energy was planned using a series of transverse slices with 1.7‐mm intervals. For each slice, the operator defined manually the contour of the area to be treated, which matched the limits of the endometriotic lesion. The Focal One software automatically dispensed the burst of the HIFU beam in order to cover the target area entirely. The same procedure was carried out for each transverse section, in order to cover the entire volume of the endometriotic lesion (Figure 2b). Once all slices had been defined, HIFU treatment could begin.

The software controlled electronically the focal distance along the acoustic axis, while the device controlled the position of the probe, moving it backwards along its longitudinal axis and rotating it on its transverse axis. The sagittal axis of the probe remained fixed during the whole procedure. The distribution and number of beams per section depended directly on the size and form of the endometriotic nodule. In order to prevent the risk of rectovaginal fistulae, a safety margin of 3 mm from the internal wall of the rectum was respected, as is recommended in the treatment of prostate cancer (Figure 2b).

### Follow‐up

All patients were contacted systematically between the 8^th^ and the 12^th^ postoperative day and asked to report any adverse effects. They were then followed up postoperatively for 6 months, undergoing clinical examinations and completing health surveys to evaluate their gynecological and intestinal symptoms and quality of life (SF‐36) at 1, 3 and 6 months. Sonographic follow‐up was scheduled at 3 and 6 months after TR‐HIFU treatment while MRI was scheduled at 6 months.

### Statistical analysis

All parameters collected were analyzed descriptively using Microsoft Excel software (Microsoft Office for Mac version 16.9, Microsoft Corp., Redmond, WA, USA). All patient data were collected and anonymized in an Excel spreadsheet. Statistical analyses and graphs were created with IBM SPSS v 24 software (IBM, Armonk, NY, USA). Means, SDs and 95% CIs were used to estimate the reliability of descriptive data. The Mann–Whitney–Wilcoxon test was used to compare pre‐ and postoperative paired data (including lesion size, and patients' VAS scores and SF‐36 questionnaire results) and to assess the influence on feasibility of whether the lesion location was rectal or sigmoid and whether the treatment was complete or partial (treatment categorized as: no treatment performed (failure), treatment of entire volume (complete) or treatment of part of the volume due to technical issues (partial)). Primary endpoint analysis was carried out following an intention‐to‐treat principle. Patients with missing data were excluded from the analysis of secondary endpoints. *P* < 0.05 was considered statistically significant. We performed no sample size or power calculation, as the upper limit on numbers for this pilot study was imposed by the ethics committee (up to 20 procedures).

## RESULTS

Between September 2015 and April 2019, 23 consecutive patients were included in this study. Follow‐up was completed in October 2019. No screened patient declined to participate. Patient characteristics are summarized in Table 1.

**Table 1:** Characteristics of patients in study investigating treatment of rectosigmoid deep infiltrating endometriosis with transrectal high‐intensity focused ultrasound (*n* = 23; *n* = 20 treated)

Characteristic	Value
Age (years)	33.4 ± 5.9 (34) [26–45]
Body mass index (kg/m^2^)	22.1 ± 3.2 (21.3) [17.3–30.4]
Gravidity	1 ± 1.3 (0) [0–5]
Parity	0.45 ± 0.8 (0) [0–2]
History of surgery for endometriosis*	7 (30.4)
Superficial peritoneal endometriosis	3 (13.0)
Endometrioma	2 (8.7)
Bladder/vesicouterine pouch	2 (8.7)
Retrocervical space/USL	2 (8.7)
Nodule location	
Mid rectum	8 (34.8); 7 treated
High rectum	12 (52.2); 12 treated
Sigmoid	3 (13.0); 1 treated

Data are given as mean ± SD (median) [range] or *n* (%).

*Some patients had more than one location for surgery.

USL, uterosacral ligaments.

The TR‐HIFU procedure was performed under spinal anesthesia in 22 (95.7%) cases. One (4.3%) patient declined spinal anesthesia so instead had general anesthesia. The mean ± SD duration of treatment was 55.6 ± 21.6 min, from insertion to removal of the rectal ultrasound probe. All patients were discharged home the day after the procedure.

**Figure 3:**
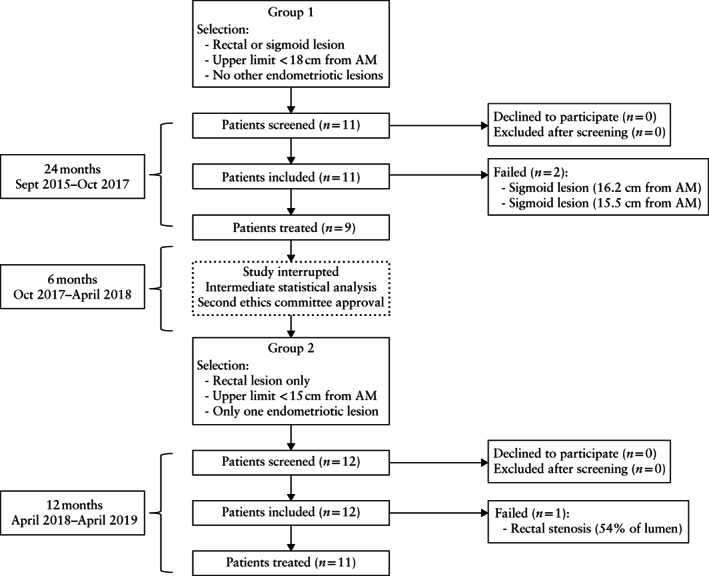
Flowchart summarizing inclusion of patients in study. AM, anal margin.

We were able to detect the endometriotic lesion in all 23 patients, giving a detection rate of 100%. Twenty of the 23 patients met all the safety requirements necessary to allow HIFU treatment, giving a feasibility rate of 87.0%. The first two treatment failures were in patients presenting a sigmoid lesion with upper limits located at 16.2 and 15.5 cm from the anal margin, respectively. The third was linked to a stenosis of the rectum estimated at 54% of the sagittal diameter of the lumen on water‐contrast MRI. Figure 3 is a flowchart summarizing the study.

The entire lesion was treated in 13 patients. The other seven patients underwent treatment of approximately 50% of the nodule. Each HIFU burst lasted 1 s and its acoustic power varied depending on the focal distance. The mean ± SD cumulative duration of HIFU was 210 ± 114 s, with delivery of mean ± SD acoustic power of 50.9 ± 11.8 W. During the six first procedures, we used intravascular ultrasound contrast (SonoVue®, Bracco, Massy, France). However, we judged that this provided contrast enhancement neither before nor after HIFU treatment. Thus, to avoid unnecessary risk, we chose not to use contrast medium for the remaining procedures.

At preoperative 3D‐RSG, the mean ± SD lesion height was 24.0 ± 6.0 mm, width was 13.2 ± 4.3 mm, thickness was 10.3 ± 5.4 mm and volume was 2.4 ± 2.5 cm^3^. At preoperative MRI, the mean ± SD lesion height was 28.2 ± 7.2 mm, width was 25.1 ± 5.9 mm, thickness was 11.9 ± 4.5 mm and volume was 4.2 ± 2.9 cm^3^.

Moderate, non‐significant reduction of the nodule mean volume was noted on 3D‐RSG both 3 and 6 months after TR‐HIFU treatment (–0.7 cm^3^, *P* = 0.16 and –0.4 cm^3^, *P* = 0.25, respectively). Similar findings were observed on postoperative MRI at 6 months, with a moderate reduction in mean nodule height (–3.3 mm, *P* = 0.1), width (–2.4 mm, *P* = 0.025) and volume (–0.2 cm^3^, *P* = 0.13), with the difference in width being the only significant finding in both imaging procedures. Nodule size and volume values, before and after TR‐HIFU treatment, on 3D‐RSG and MRI, are reported in Table 2.

**Table 2:** Comparison of preoperative (M0) and 3‐month (M3) and 6‐month (M6) postoperative 3D rectosonography findings and magnetic resonance imaging (MRI) findings preoperatively and at M6 (paired data) in 20 patients who underwent transrectal high‐intensity focused ultrasound therapy for rectosigmoid deep infiltrating endometriosis

	3D Rectosonography					MRI		
Lesion dimension	M0	M3	M3 − M0	M6	M6 − M0	M0	M6	M6 − M0
Height (mm)	24.0 ± 6.0	20.9 ± 8.5	–3.1	20.9 ± 5.1	–3.1	28.2 ± 7.2	24.9 ± 8.4	–3.3
Width (mm)	13.2 ± 4.3	9.4 ± 3.5	–3.8	7.7 ± 2.6	–5.5*	25.1 ± 5.9	22.7 ± 6.0	–2.4*
Thickness (mm)	10.3 ± 5.4	7.8 ± 8.8	–2.5	10.1 ± 6.1	–0.2	11.9 ± 4.5	10.4 ± 4.9	–1.5
Volume (cm^3^)	2.4 ± 2.5	1.7 ± 1.3	–0.7	2.0 ± 1.4	–0.4	4.2 ± 2.9	4.0 ± 2.4	–0.2

Values are mean ± SD or difference.

Preoperative *vs* postoperative (Mann–Whitney–Wilcoxon test):

* *P* < 0.05.

**Figure 4:**
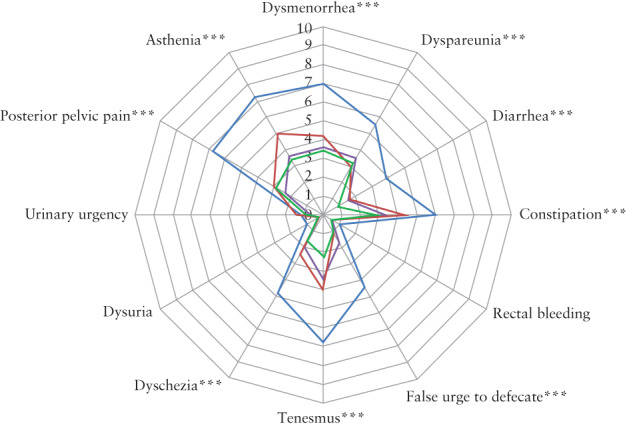
Spiderweb plot comparing symptom intensity according to visual analog scale, before (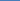), and 1 month (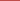), 3 months (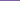) and 6 months (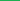) after, transrectal high‐intensity focused ultrasound therapy (TR‐HIFU) for rectosigmoid deep infiltrating endometriosis. Paired data comparing pre‐ *vs* post‐TR‐HIFU: *P ≤ 0.05 at one follow‐up examination; **P ≤ 0.05 at two follow‐up examinations; ***P ≤ 0.05 at all follow‐up examinations.

Gynecological and intestinal symptoms preoperatively and their evolution postoperatively are summarized in Table 3 and Figure 4. At 1 month, 3 months and 6 months postoperatively, there was a significant reduction compared with preoperatively in VAS score for dysmenorrhea, dyspareunia, diarrhea, constipation, false urge to defecate, tenesmus, dyschezia, posterior pelvic pain and asthenia, while there was no significant difference for rectal bleeding and urinary symptoms related to endometriosis. No significant differences in symptoms between 1‐ and 6‐months postprocedure were observed.

**Table 3:** Comparison of preoperative (M0) and 1‐month (M1), 3‐month (M3) and 6‐month (M6) postoperative symptom intensity (paired data) in 20 patients who underwent transrectal high‐intensity focused ultrasound therapy for rectosigmoid deep infiltrating endometriosis

Parameter	Preop VAS: M0	Postop VAS: M1	Delta VAS: M1 − M0	*P* *	Postop VAS: M3	Delta VAS: M3 − M0	*P* *	Postop VAS: M6	Delta VAS: M6 − M0	*P* *
Dysmenorrhea	7.0 ± 3.0	4.2 ± 2.9	–2.8	0.003	3.6 ± 3.3	–3.4	0.002	3.4 ± 3.3	–3.6	0.004
	(7.5) [0–10]	(5.5) [0–7]			(3.5) [0–9]			(3.0) [0–9]		
Dyspareunia	5.6 ± 3.2	2.9 ± 3.2	–2.7	0.008	3.5 ± 2.8	–2.1	0.012	3.2 ± 2.9	–2.4	0.006
	(7.0) [0–10]	(2.5) [0–9]			(4.0) [0–9]			(3.5) [0–9]		
Diarrhea	3.9 ± 3.8	1.6 ± 2.4	–2.3	0.008	1.5 ± 2.5	–2.4	0.012	0.9 ± 1.5	–3.0	0.006
	(5.0) [0–10]	(0.0) [0–7]			(0.0) [0–7]			(0.0) [0–5]		
Constipation	5.9 ± 3.5	4.4 ± 3.2	–1.5	0.024	3.4 ± 2.8	–2.5	0.015	2.9 ± 3.2	–3.0	0.002
	(6.5) [0–10]	(4.0) [0–10]			(3.0) [0–9]			(2.0) [0–9]		
Rectal	1.1 ± 2.8	0.5 ± 1.9	–0.6	0.102	0.6 ± 2.4	–0.5	0.414	0.5 ± 1.9	–0.6	0.102
bleeding	(0.0) [0–9]	(0.0) [0–8]			(0.0) [0–10]			(0.0) [0–8]		
False urge to	4.4 ± 3.7	1.2 ± 2.3	–3.2	0.009	1.7 ± 2.8	–2.7	0.026	1.1 ± 2.3	–3.3	0.007
defecate	(4.5) [0–10]	(0.0) [0–7]			(0.0) [0–9]			(0.0) [0–7]		
Tenesmus	6.8 ± 3.5	3.9 ± 3.1	–2.9	0.016	3.5 ± 3.4	–3.3	0.021	2.2 ± 2.9	–4.6	0.015
	(8.0) [0–10]	(4.0) [0–8]			(3.0) [0–10]			(2.0) [0–9]		
Dyschezia	4.8 ± 3.8	2.4 ± 3.0	–2.4	0.005	1.9 ± 2.6	–2.9	0.005	1.6 ± 2.3	–3.2	0.003
	(6.5) [0–10]	(1.0) [0–7]			(0.5) [0–9]			(0.0) [0–7]		
Dysuria	1.0 ± 2.5	0.2 ± 0.7	–0.8	0.102	0.3 ± 1.2	–0.7	0.109	0.2 ± 0.7	–0.8	0.109
	(0.0) [0–9]	(0.0) [0–3]			(0.0) [0–5]			(0.0) [0–3]		
Urinary urgency	1.2 ± 2.7	1.4 ± 1.8	+ 0.2	0.574	0.7 ± 1.6	–0.5	0.465	0.9 ± 2.1	–0.3	0.683
	(0.0) [0–8]	(0.0) [0–5]			(0.0) [0–5]			(0.0) [0–8]		
Posterior	6.7 ± 2.6	3.1 ± 3.0	–3.6	0.002	2.2 ± 2.8	–4.5	0.002	2.9 ± 2.9	–3.8	0.002
pelvic pain	(7.5) [0–10]	(2.5) [0–7]			(1.0) [0–9]			(3.0) [0–8]		
Asthenia	7.2 ± 2.5	4.9 ± 2.3	–2.3	0.002	3.6 ± 3.0	–3.6	0.003	3.4 ± 3.1	–3.8	0.002
	(8.0) [0–10]	(5.0) [0–7]			(3.0) [0–9]			(3.0) [0–8]		

Visual analog scale (VAS) values are given as mean ± SD (median) [range].

* Mann–Whitney–Wilcoxon test: Preoperative (Preop) *vs* Postoperative (Postop) VAS.

**Figure 5:**
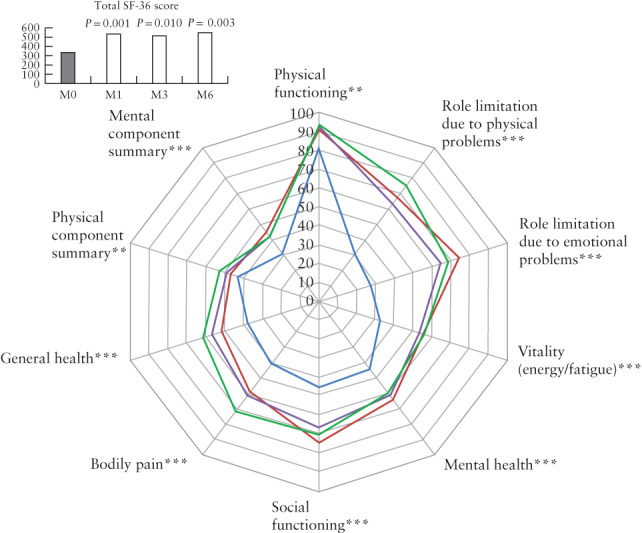
Spiderweb plot comparing Medical Outcomes Study 36‐item short‐form survey (SF‐36) items scores and SF‐36 total score before (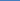), and 1 month (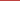), 3 months (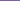) and 6 months (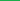) after, transrectal high‐intensity focused ultrasound therapy (TR‐HIFU) for rectosigmoid deep infiltrating endometriosis. Paired data comparing pre‐ *vs* post‐TR‐HIFU: *P ≤ 0.05 at one follow‐up examination; **P ≤ 0.05 at two follow‐up examinations; ***P ≤ 0.05 at all follow‐up examinations.

The SF‐36 score results are presented in Table 4 and Figure 5. At 1 month postoperatively, all components of the SF‐36 score were significantly improved relative to preoperatively, except for physical functioning. At 3 and 6 months, all components of the SF‐36 score were significantly improved relative to preoperatively. We observed a statistically significant improvement, at 1 postoperative month, in the MCS of 14.5 points (+ 47.1%; *P* = 0.001) and in the overall SF‐36 score of + 198 points (+ 58.2%; *P* = 0.001), and at 6 postoperative months of + 10.9 points (+ 35.4%; *P* = 0.017) and of + 217 points (+ 63.8%; *P* = 0.003), respectively. The PCS score showed a trend towards improvement at 1 postoperative month, which was then significant at 3 and 6 postoperative months, with + 5.9 points (+ 12.6%; *P* = 0.017) and + 9.3 points (+ 21.7%; *P* = 0.002), respectively.

**Table 4:** Comparison of preoperative (M0) and 1‐month (M1), 3‐month (M3) and 6‐month (M6) postoperative Medical Outcomes Study 36‐item short‐form survey (SF‐36) scores (paired data), which reflect quality of life, in 20 patients who underwent transrectal high‐intensity focused ultrasound therapy for rectosigmoid deep infiltrating endometriosis

Parameter	Preop SF‐36 score: M0	Postop SF‐36 score: M1	Delta SF‐36 score: M1 − M0	*P* *	Postop SF‐36 score: M3	Delta SF‐36 score: M3 − M0	*P* *	Postop SF‐36 score: M6	Delta SF‐36 score: M6 − M0	*P* *
Physical functioning	81.2 ± 19.5 (85) [40–100]	90.0 ± 8.8 (95) [75–100]	+ 8.8	0.062	92.1 ± 9.0 (90) [75–100]	+ 10.9	0.007	93.8 ± 8.0 (100) [75–100]	+ 12.6	0.011
Role limitation:										
Physical	30.9 ± 31.3 (25) [0–100]	67.6 ± 37.3 (75) [0–100]	+ 36.7	0.021	63.2 ± 39.6 (75) [0–100]	+ 32.3	0.032	75.0 ± 37.5 (100) [0–100]	+ 44.1	0.010
Emotional	27.5 ± 35.8 (0) [0–100]	74.5 ± 30.1 (67) [0–100]	+ 47	0.004	64.7 ± 46.4 (100) [0–100]	+ 37.2	0.015	68.6 ± 39.9 (100) [0–100]	+ 41.1	0.010
Vitality	32.9 ± 15.1 (35) [5–60]	55.3 ± 17.1 (60) [20–80]	+ 22.4	0.004	53.5 ± 22.6 (60) [5–80]	+ 20.6	0.009	55.9 ± 23.7 (65) [15–95]	+ 23	0.007
Mental health	44.2 ± 20.3 (44) [12–92]	64.2 ± 18.8 (56) [20–96]	+ 20	0.001	61.2 ± 19.4 (64) [28–84]	+ 17	0.017	59.8 ± 21.5 (64) [24–88]	+ 15.6	0.019
Social functioning	45.6 ± 18.2 (50) [13–75]	75.0 ± 17.7 (75) [38–100]	+ 29.4	0.002	66.9 ± 25.0 (75) [13–100]	+ 21.3	0.019	70.6 ± 22.9 (75) [38–100]	+ 25	0.013
Bodily pain	40.4 ± 14.7 (45) [13–68]	59.3 ± 22.7 (58) [23–90]	+ 18.9	0.009	61.5 ± 27.3 (70) [23–90]	+ 21.1	0.013	71.8 ± 20.1 (78) [35–100]	+ 31.4	0.001
General health	37.6 ± 18.0 (35) [10–75]	51.8 ± 16.7 (50) [20–85]	+ 14.2	0.009	56.8 ± 19.9 (60) [15–95]	+ 19.2	0.005	61.5 ± 22.1 (65) [20–100]	+ 23.9	0.004
PCS (%)	42.9 ± 6.7 (43) [29–55]	46.8 ± 5.3 (48) [31–53]	+ 3.9	0.193	48.8 ± 6.8 (51) [37–60]	+ 5.9	0.017	52.2 ± 6.2 (51) [42–64]	+ 9.3	0.002
MCS (%)	30.8 ± 10.5 (28) [15–55]	45.3 ± 9.4 (48) [22–60]	+ 14.5	0.001	41.9 ± 12.3 (45) [17–56]	+ 11.1	0.020	41.7 ± 12.8 (47) [21–57]	+ 10.9	0.017
Total SF‐36 score	340 ± 121 (355) [130–592]	538 ± 129 (561) [248–674]	+ 198	0.001	520 ± 163 (518) [197–720]	+ 180	0.010	557 ± 156 (629) [300–761]	+ 217	0.003

SF‐36 scores are given as mean ± SD (median) [range].

* Mann–Whitney–Wilcoxon test: Preoperative (Preop) *vs* Postoperative (Postop).

MCS, mental component summary; PCS, physical component summary.

**Figure 6:**
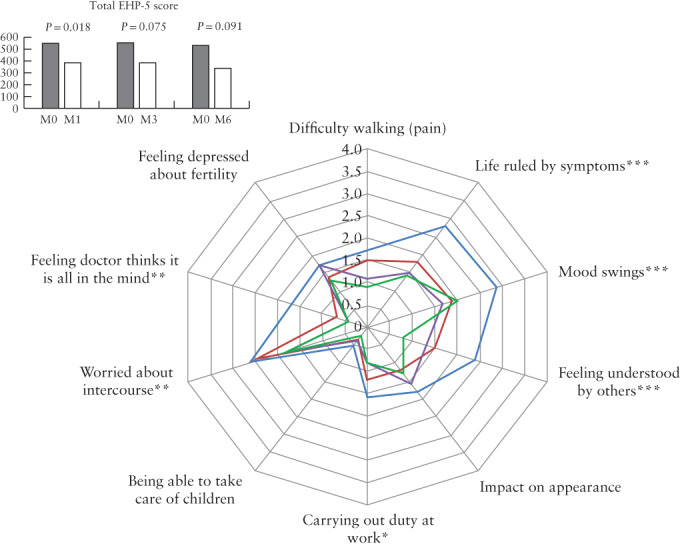
Spiderweb plot comparing between different components of endometriosis health profile short‐version (EHP‐5) score before (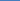), and 1 month (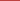), 3 months (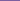) and 6 months (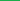) after, transrectal high‐intensity focused ultrasound therapy (TR‐HIFU) for rectosigmoid deep infiltrating endometriosis. Paired data comparing pre‐ *vs* post‐TR‐HIFU: *P ≤ 0.05 at one follow‐up examination; **P ≤ 0.05 at two follow‐up examinations; ***P ≤ 0.05 at all follow‐up examinations.

**Figure 7:**
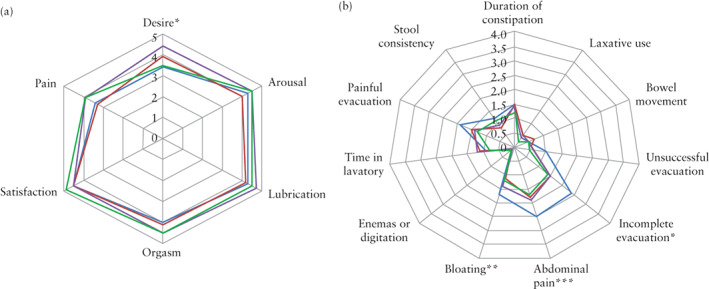
Spiderweb plot comparing between results of female sexual function index (FSFI) score (sexuality‐related quality of life) (a) and symptom scoring system for constipation (KESS) score (constipation‐related quality of life) (b), before (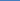), and 1 month (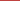), 3 months (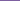) and 6 months (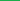) after, transrectal high‐intensity focused ultrasound therapy (TR‐HIFU) for rectosigmoid deep infiltrating endometriosis. Paired data comparing pre‐ *vs* post‐TR‐HIFU: *P ≤ 0.05 at one follow‐up examination; **P ≤ 0.05 at two follow‐up examinations; ***P ≤ 0.05 at all follow‐up examinations.

For the last 11 patients treated, who completed additional questionnaires, the EHP‐5 scores showed significant improvement in several respects at 6 postoperative months (Figure 6 and Table S2). However, the improvement in total EHP‐5 score was not significant at 6 months (*P* = 0.091). The FSFI sexuality score, for these same patients, did not show significant improvement over time, but there was a trend towards improvement in pain score at 6 months (3.9 *vs* 3.4, + 0.5, *P* = 0.22) (Figure 7a, Table S3). As observed for constipation symptoms on the VAS score, the KESS score showed a global improvement in constipation‐related quality of life at 6 postoperative months (10.1 *vs* 14.1, *P* = 0.026) (Figure 7b, Table S4).

No major complication scoring higher than Grade II (according to the Clavien–Dindo classification) occurred during treatment or in the follow‐up period. Among the 20 patients treated, 14 (70%) reported minor or moderate adverse events. The main side effects reported were minor rectal bleeding in four (20%) cases and moderate pain in various locations, including abdomen, pelvis, back and vagina, in five (25%) cases. Other side effects included moderate anal pain in five (25%) cases and abdominal bloating, which lasted until the 12^th^ postoperative day, in two (10%). One patient experienced cerebrospinal fluid leakage related to spinal anesthesia on day 1 and was treated with a blood patch. All adverse events potentially related to the procedure, with their classification and management, are reported in Table 5.

**Table 5:** Summary of overall adverse events potentially related to transrectal high‐intensity focused ultrasound therapy for rectosigmoid deep infiltrating endometriosis (*n* = 20)

Adverse event	Events (*n*)	Subjects (*n* (%))	Treatment	Clavien–Dindo grade^34^
Overall	41	14 (70)		
Gastrointestinal	21	11 (55)		
Constipation	6	6 (30)	None/laxative	I
Anal pain	5	5 (25)	None/analgesic	I
Rectal bleeding	4	4 (20)	None	I
Abdominal distension	2	2 (10)	None	I
Abdominal pain	1	1 (5)	Analgesic	I
Diarrhea	1	1 (5)	Antidiarrhea	I
Rectal tenesmus	1	1 (5)	Analgesic	I
Anorectal discomfort	1	1 (5)	None	I
Reproductive system	8	6 (30)		
Pelvic pain	3	3 (15)	Analgesic	I
Metrorrhagia	2	2 (10)	None	I
Vulvovaginal pain	1	1 (5)	None	I
Dyspareunia	1	1 (5)	None	I
Vaginal discharge	1	1 (5)	None	I
General/musculoskeletal	7	6 (30)		
Asthenia	2	2 (10)	None	I
Back pain	2	2 (10)	Analgesic	I
Flank pain	1	1 (5)	Analgesic	I
Weight decrease	1	1 (5)	None	I
Gait disturbance	1	1 (5)	None	I
Renal/urinary	3	3 (15)		
Pollakiuria	1	1 (5)	None	I
Urinary tract discomfort	1	1 (5)	None	I
Micturition disorder	1	1 (5)	None	I
Nervous system disorder	1	1 (5)		
Transient lower‐limb hypoesthesia	1	1 (5)	None	I
Injury and procedural complications	1	1 (5)		
Cerebrospinal fluid leakage	1	1 (5)	Blood patch	II

## DISCUSSION

To our knowledge, this is the first clinical trial reporting TR‐HIFU therapy in patients presenting with rectosigmoid DIE. We demonstrated the feasibility of using the Focal One device to detect these lesions (100%) as well as for their treatment (87%). No major complication was reported during the 6‐month period post‐treatment. We observed a significant improvement in gynecological and intestinal symptoms as well as improvement in the quality of life of affected patients, despite only small and insignificant morphological modifications on imaging examination.

A morphological modification has, however, been described following HIFU treatment in prostate cancer and, more recently, in parietal endometriotic nodules^15^, ^35^. Luo *et al*.^15^ reported a significant diminution in volume of parietal lesions at 6 months after surgery compared with the initial volume (1.33 ± 0.31 cm^3^ *vs* 2.80 ± 0.12 cm^3^, *P* < 0.05). We propose two possible explanations for the absence of decreased volume in our study: the fact that several (35%) patients underwent only partial treatment and our decision to use a reduced HIFU dose for reasons of safety. However, morphological modification is not the primary objective of the treatment; it is simply a way of demonstrating that the energy is indeed concentrated in the targeted lesion. The main objective of TR‐HIFU therapy is to relieve the symptoms of patients, regardless of any change in nodule size.

Although all endometriotic lesions were visualized preoperatively using the diagnostic probe, in three cases, treatment could not be carried out and in seven cases treatment was only partial. These difficulties were related mainly to the morphology of the Focal One probe. The device was designed for prostate cancer treatment, with the prostate being located just above the anal canal and facing the lower rectum^12^. In contrast, rectosigmoid endometriotic lesions are located above the posterior vaginal cuff, at the mid‐rectum and above. Thus, often, maneuvering of the probe was restricted by the patient's sacrum. For future studies, it seems logical to limit inclusion to patients who could be treated fully (excluding those with a sigmoid lesion located > 15 cm from the anal margin and those with stenotic lesions) and also to suggest structural modification of the TR‐HIFU probe. Similarly, the 3‐mm safety margin did not allow complete treatment of nodules invading the mucosae. However, we believe that partial treatment can improve a patient's quality of life. Larger studies are required to evaluate the impact of partial treatment on efficacy of treatment and the chance of recurrence.

Although this was a feasibility study, we report a significant impact of RT‐HIFU treatment for rectal DIE in terms of improvement in quality of life and symptoms 1 month postoperatively, with ongoing improvement within the follow‐up period of 6 months postoperatively. These results are consistent with those published on HIFU treatment of abdominal wall endometriosis, which also showed a significant improvement in postoperative pain^15^, ^16^. According to Guan and Xu^36^, HIFU treatment can lead to necrosis of the cells responsible for the loss of function of the ectopic endometrium, thereby resulting in attenuation of symptoms. This non‐controlled study was not designed to prove clinical efficiency and the subjective aspect involved in the estimation of symptoms and quality of life is one of its limitations. The possibility of a placebo effect cannot be excluded, particularly because we did not observe a significant reduction in nodule size following treatment. We chose to use a VAS and previously validated questionnaires to assess quality of life (SF‐36, EHP‐5, FSFI and KESS scores)^24^, ^25^ in order to limit this subjectivity.

One of the reasons we tested the efficiency of HIFU treatment of rectosigmoid endometriosis was to find a less morbid alternative compared with the standard treatment, surgery. We observed no major complications and only minor adverse effects during the 6‐month follow‐up period of our study, consistent with the findings of Luo *et al*.^15^ and Zhu *et al*.^16^, who likewise did not report any severe complications in the 6‐month period following use of HIFU in the management of parietal endometriotic nodules. This minimal morbidity was also observed following TR‐HIFU treatment for localized prostate cancer, while the ultrasound dose used was twice the established dose used in our protocol^35^, ^37^, ^38^; rectourethral fistulae were rare, with a rate varying between 0.13% and 0.6%, and tended to be less likely (0.2% rate) following treatment with the latest‐generation HIFU device, Focal One.

To conclude, based on this study of a small number of patients, treatment by TR‐HIFU of rectosigmoid endometriotic lesions seems to be feasible and safe. The limited morbidity and significant improvement of intestinal and gynecological symptoms, as well as in the quality of life, observed in this study is very promising. However, the safety and objective clinical efficacy in the short‐ and long‐term remains to be proven. Further research is required with larger numbers of patients to confirm these findings and a HIFU dose escalation study should be considered.

## Supporting information


**Table S1** Main characteristics of high‐intensity focused ultrasound probe
**Table S2** Endometriosis health profile short‐version (EHP‐5) score
**Table S3** Female sexual function index (FSFI) score
**Table S4** Symptom scoring system for constipation (KESS) score
