# Supplementary material for: Transrectal high‐intensity focused ultrasound (HIFU) for management of rectosigmoid deep infiltrating endometriosis: results of Phase‐I clinical trial
Source: Ultrasound Obstet Gynecol. 2020 Sep 1;56(3):431–42. doi: 10.1002/uog.21937 (PMC7496183; doi:10.1002/uog.21937)
Supplement: Supplementary file 1 — Table S1 Main characteristics of high‐intensity focused ultrasound probe Table S2 Endometriosis health profile short‐version (EHP‐5) score Table S3 Female sexual function index (FSFI) score Table S4 Symptom scoring system for constipation (KESS) score [file UOG-56-431-s001.docx]

**Supplementary table 1. Main characteristics of HIFU probe**

| **Frequency of therapeutic transducer** | 3 MHz |
| --- | --- |
| **Diameter** | 57.5mm |
| **Natural focal distance** | 60mm |
| **Truncation** | 35mm |
| **Opening diameter** | 24.8mm |
| **Focal width along longitudinal dimension** | axis X: 0.58mm (-6dB) |
| **Focal width along transversal dimension** | axis Y: 1mm (-6dB) |
| **Focal width along acoustic direction** | axis Z : 6.4mm (-6dB) at F= 60 mm |

**Supplementary table 2: EHP 5 score**

| EHP5 item | mean ± SD (médiane) [Min-Max] | p-value (vs M0) |
| --- | --- | --- |
| **Q11**: Difficulty to walk (pain) (N=11) |  |  |
| M0 | 1.7 ± 1.2 (2) [0-3] |  |
| M1 | 1.5 ± 1.1 (1) [0-3] | p=0.527 |
| M3 | 1.1 ± 1.0 (1) [0-3] | p=0.084 |
| M6 | 0.9 ± 0.9 (1) [0-2] | p=0.101 |
| **Q12:** Life ruled by symptoms (N=11) |  |  |
| M0 | 2.8 ± 0.6 (3) [2-4] |  |
| M1 | 1.8 ± 1.0 (2) [0-3] | **p=0.018** |
| M3 | 1.5 ± 1.0 (2) [0-3] | **p=0.008** |
| M6 | 1.4 ± 1.1 (1) [0-4] | **p=0.008** |
| **Q13 :** Mood swings (N=11) |  |  |
| M0 | 2.9 ± 0.8 (3) [1-4] |  |
| M1 | 1.9 ± 0.5 (2) [1-3] | **p=0.009** |
| M3 | 1.7 ± 0.9 (2) [0-3] | **p=0.018** |
| M6 | 2.0 ± 0.9 (2) [1-3] | **p=0.020** |
| **Q14:** Feeling understood by others (N=11) |  |  |
| M0 | 2.4 ± 0.9 (2) [1-4] |  |
| M1 | 1.5 ± 1.2 (1) [0-4] | **p=0.030** |
| M3 | 1.3 ± 1.2 (1) [0-4] | **p=0.025** |
| M6 | 0.8 ± 0.9 (1) [0-2] | **p=0.007** |
| **Q15:** Impact on appearance (N=11) |  |  |
| M0 | 1.8 ± 1.3 (2) [0-4] |  |
| M1 | 1.2 ± 1.2 (1) [0-3] | p=0.068 |
| M3 | 1.6 ± 1.0 (2) [0-3] | p=0.726 |
| M6 | 1.3 ± 1.3 (1) [0-3] | p=0.161 |
| **Q21:** Carrying out duty at work (N=11) |  |  |
| M0 | 1.6 ± 1.0 (2) [0-3] |  |
| M1 | 1.2 ± 0.9 (1) [0-3] | p=0.236 |
| M3 | 0.8 ± 1.1 (0) [0-3] | **p=0.030** |
| M6 | 0.8 ± 1.3 (0) [0-4] | p=0.102 |
| **Q22:** Being able to take care of children (N=11) |  |  |
| M0 | 0.5 ± 1.0 (0) [0-3] |  |
| M1 | 0.3 ± 0.6 (0) [0-2] | p=0.180 |
| M3 | 0.4 ± 0.8 (0) [0-2] | p=0.157 |
| M6 | 0.2 ± 0.4 (0) [0-1] | p=0.102 |
| **Q23:** Worried about intercourses (N=11) |  |  |
| M0 | 2.6 ± 1.2 (3) [1-4] |  |
| M1 | 2.5 ± 1.6 (3) [0-4] | p=0.317 |
| M3 | 1.8 ± 1.6 (2) [0-4] | **p=0.007** |
| M6 | 1.8 ± 1.4 (2) [0-4] | **p=0.014** |
| **Q24:** Feeling doctor think it is all in mind (N=9) |  |  |
| M0 | 1.6 ± 1.3 (2) [0-3] |  |
| M1 | 0.7 ± 1.0 (0) [0-3] | **p=0.071** |
| M3 | 0.4 ± 0.5 (0) [0-1] | **p=0.028** |
| M6 | 0.4 ± 0.5 (0) [0-1] | **p=0.028** |
| **Q25:** Feeling depressed about fertility (N=9) |  |  |
| M0 | 1.7 ± 1.1 (2) [0-3] |  |
| M1 | 1.4 ± 1.6 (1) [0-4] | p=0.595 |
| M3 | 1.7 ± 0.9 (2) [0-3] | p=1.000 |
| M6 | 1.3 ± 1.5 (1) [0-4] | p=0.581 |
| **EHP-5 : Total score** (N=6) |  |  |
| M0 | 538 ± 171 (550) [275-775] |  |
| M1 | 371 ± 102 (388) [225-475] | **p=0.043** |
| M3 | 383 ± 127 (388) [225-550] | p=0.138 |
| M6 | 333 ± 184 (338) [125-600] | p=0.116 |
|  |  |  |

**Supplementary Table 3 : FSFI score**

| FSFI item | mean ± SD (médian) [Min-Max] | p-value (vs M0) |
| --- | --- | --- |
|  |  |  |
| **Desire score** (N=11) |  |  |
| pré-op | 3.4 ± 2.0 (3) [1-6] |  |
| 1 mois | 3.9 ± 1.6 (4) [1-6] | p=0.131 |
| 3 mois | 4.4 ± 1.4 (5) [2-6] | **p=0.031** |
| 6 mois | 3.5 ± 1.7 (4) [1-5] | p=0.719 |
|  |  |  |
| **Arousal score** (N=11) |  |  |
| pré-op | 4.3 ± 1.4 (5) [2-6] |  |
| 1 mois | 4.0 ± 2.3 (5) [0-6] | p=0.606 |
| 3 mois | 4.5 ± 1.6 (5) [1-6] | p=0.518 |
| 6 mois | 4.5 ± 1.6 (5) [1-6] | p=0.720 |
|  |  |  |
| **Lubrification score** (N=11) |  |  |
| pré-op | 4.3 ± 1.7 (5) [2-6] |  |
| 1 mois | 4.2 ± 2.5 (6) [0-6] | p=0.915 |
| 3 mois | 4.7 ± 1.7 (6) [1-6] | p=0.157 |
| 6 mois | 4.5 ± 1.7 (5) [1-6] | p=0.480 |
|  |  |  |
| **Orgasm score** (N=11) |  |  |
| pré-op | 4.0 ± 1.7 (4) [1-6] |  |
| 1 mois | 4.1 ± 2.5 (5) [0-6] | p=0.886 |
| 3 mois | 4.5 ± 1.9 (5) [0-6] | p=0.509 |
| 6 mois | 4.5 ± 1.3 (5) [2-6] | p=0.262 |
|  |  |  |
| **Satisfaction score** (N=11) |  |  |
| pré-op | 4.5 ± 1.6 (5) [2-6] |  |
| 1 mois | 4.5 ± 2.5 (6) [0-6] | p=0.916 |
| 3 mois | 4.5 ± 2.2 (6) [0-6] | p=0.932 |
| 6 mois | 4.9 ± 1.5 (5) [1-6] | p=0.234 |
|  |  |  |
| **Pain score** (N=11) |  |  |
| pré-op | 3.4 ± 1.6 (3) [1-6] |  |
| 1 mois | 3.3 ± 2.1 (3) [0-6] | p=0.763 |
| 3 mois | 3.9 ± 1.7 (4) [1-6] | p=0.196 |
| 6 mois | 3.9 ± 1.6 (4) [1-6] | p=0.223 |
|  |  |  |
| **FSFI Total score** (N=11) |  |  |
| pré-op | 23.8 ± 8.6 (27) [11-33] |  |
| 1 mois | 24.0 ± 12.7 (29) [1-35] | p=0.646 |
| 3 mois | 26.5 ± 8.9 (31) [5-35] | p=0.284 |
| 6 mois | 25.8 ± 8.1 (29) [7-35] | p=0.504 |
|  |  |  |

**Supplementary table 4 : KESS score**

| KESS item | Time of evaluation | mean ± SD (médian) [Min-Max] | p-value (vs M0) |
| --- | --- | --- | --- |
| **Q01** (N=11 | M0 | 1.5 ± 1.4 (1) [0-4] |  |
| Duration of | M1 | 1.5 ± 1.1 (1) [0-4] | p=0.783 |
| constipation | M3 | 1.4 ± 1.2 (1) [0-4] | p=0.705 |
|  | M6 | 1.2 ± 1.2 (1) [0-3] | p=0.680 |
| **Q02**(N=11) | M0 | 0.5 ± 1.0 (0) [0-3] |  |
| Laxative use | M1 | 0.5 ± 1.0 (0) [0-3] | p=1.000 |
|  | M3 | 0.4 ± 0.7 (0) [0-2] | p=0.564 |
|  | M6 | 0.2 ± 0.6 (0) [0-2] | p=0.180 |
| **Q03** (N=11) | M0 | 0.5 ± 0.7 (0) [0-2] |  |
| Bowel movement | M1 | 0.7 ± 0.9 (0) [0-2] | p=0.157 |
|  | M3 | 0.5 ± 0.7 (0) [0-2] | p=1.000 |
|  | M6 | 0.5 ± 0.7 (0) [0-2] | p=1.000 |
| **Q04** (N=11) | M0 | 1.0 ± 0.8 (1) [0-2] |  |
| Unsuccessful | M1 | 0.5 ± 0.8 (0) [0-2] | p=0.084 |
| evacuation | M3 | 0.6 ± 0.8 (0) [0-2] | p=0.102 |
|  | M6 | 0.5 ± 0.5 (1) [0-1] | p=0.059 |
| **Q05** (N=11) | M0 | 2.4 ± 1.4 (3) [0-4] |  |
| Incomplete | M1 | 1.5 ± 1.4 (1) [0-4] | p=0.074 |
| evacuation | M3 | 1.5 ± 1.1 (2) [0-4] | **p=0.054** |
|  | M6 | 1.4 ± 1.1 (1) [0-3] | **p=0.039** |
| **Q06** (N=11) | M0 | 2.5 ± 1.0 (2) [1-4] |  |
| Abdominal pain | M1 | 1.8 ± 0.9 (2) [1-3] | **p=0.035** |
|  | M3 | 1.9 ± 0.9 (2) [0-3] | **p=0.034** |
|  | M6 | 1.7 ± 0.8 (2) [1-3] | **p=0.020** |
| **Q07** (N=11) | M0 | 1.7 ± 0.9 (1) [1-3] |  |
| Bloating | M1 | 1.1 ± 0.7 (1) [0-2] | **p=0.038** |
|  | M3 | 1.4 ± 0.5 (1) [1-2] | p=0.102 |
|  | M6 | 1.2 ± 0.6 (1) [0-2] | **p=0.034** |
| **Q08** (N=11) | M0 | 0.1 ± 0.3 (0) [0-1] |  |
| Enemas/digitation | M1 | 0.1 ± 0.3 (0) [0-1] | p=1.000 |
|  | M3 | 0.1 ± 0.3 (0) [0-1] | p=1.000 |
|  | M6 | 0.2 ± 0.4 (0) [0-1] | p=0.317 |
| **Q09** (N=11) | M0 | 0.9 ± 0.5 (1) [0-2] |  |
| Time in lavatory | M1 | 1.1 ± 0.9 (1) [0-3] | p=0.317 |
|  | M3 | 1.2 ± 0.8 (1) [0-3] | p=0.083 |
|  | M6 | 0.8 ± 0.6 (1) [0-2] | p=0.564 |
| **Q10** (N=11) | M0 | 1.9 ± 1.3 (2) [0-3] |  |
| Painful | M1 | 1.5 ± 1.0 (1) [0-3] | p=0.163 |
| evacuation | M3 | 1.4 ± 0.9 (1) [0-3] | p=0.196 |
|  | M6 | 1.3 ± 0.6 (1) [0-2] | p=0.191 |
| **Q11** (N=11) | M0 | 1.2 ± 1.0 (1) [0-3] |  |
| Stool consistency | M1 | 0.8 ± 1.0 (1) [0-3] | p=0.046 |
|  | M3 | 0.9 ± 0.8 (1) [0-3] | p=0.257 |
|  | M6 | 1.1 ± 0.8 (1) [0-3] | p=0.564 |
| **KESS: Total score** (N=11) | M0 | 14.1 ± 6.4 (13) [2-22] |  |
|  | M1 | 11.1 ± 5.4 (10) [1-19] | **p=0.028** |
|  | M3 | 11.3 ± 4.7 (11) [3-19] | **p=0.021** |
|  | M6 | 10.1 ± 3.8 (10) [5-15] | **p=0.026** |
|  |  |  |  |
